# Supplementary material for: What Do We See in Spectra?: Assignment of High-Intensity Peaks of Cutibacterium and Staphylococcus Spectra of MALDI-TOF Mass Spectrometry by Interspecies Comparative Proteogenomics
Source: Microorganisms. 2021 Jun 8;9(6):1243. doi: 10.3390/microorganisms9061243 (PMC8227259; doi:10.3390/microorganisms9061243)

Supplemental material

What do we see in spectra?: Assignment of high-intensity peaks of *Cutibacterium* and *Staphylococcus* spectra of MALDI-TOF mass spectrometry by inter-species comparative proteogenomics

Itaru Dekio,[1] Yuki Sugiura,[1] Susumu Hamada-Tsutsumi,[2] Yoshiyuki Murakami,[3] Hiroto Tamura,[2] Makoto Suematsu[1]

1. Department of Biochemistry & Integrative Medical Biology, School of Medicine, Keio University, 35 Shinanomachi, Shinjuku-ku, Tokyo 160-8582, Japan

2. Department of Environmental Bioscience, Meijo University, 1-501 Shiogamaguchi, Tenpaku-ku, Nagoya, Aichi 468-8502, Japan

3. Seikakai Mildix Skin Clinic, 3rd Floor, 3-98 Senju, Adachi-ku, Tokyo 120-0034, Japan

*Correspondence: Itaru Dekio, M.D., Ph.D.

[Present address]

Department of Dermatology, School of Medicine, The Jikei University

3-25-8 Nishi-shinbashi, Minato-ku, Tokyo 105-8461, Japan

Tel: +81-3-3433-1111 ext 3341, Fax: +81-3-5401-0125.

E-mail: i@skinmicrobe.com

Table S1. List of non-ribosomal proteins detected in in-gel analysis of five strains of genus *Cutibacterium*. Proteins with molecular weight of 15 kD or below are shown. acn IA_1: *C. acnes* subsp. *acnes* type IA_1, acn IB: *C. acnes* subsp. *acnes* type IB, acn II: *C. acnes* subsp. *defendens* type II, acn III: *C. acnes* subsp. *elongatum* type III, mod: *C. modestum*. + indicates detection.

Protein name	MW (kD)	acn IA_1	acn IB	acn II	acn III	mod
Uncharacterized protein	5				+	
CsbD-like protein	7	+	+	+	+	+
Phosphotransferase EIIB	7		+		+	
Methylmalonyl-CoA carboxytransferase subunit 12S	8	+	+	+		+
DUF3107/ATP-binding protein	8				+	
Acyl carrier protein	8-9	+	+	+	+	+
DNA-binding protein HU	9	+		+	+	
Uncharacterized protein	9	+				
PspC domain-containing protein	9-10	+	+		+	+
10 kD chaperonin	10	+	+	+	+	+
Uncharacterized protein	10				+	
DUF4235 domain-containing protein	10		+			
Biotin carboxyl carrier protein of methylmalonyl-CoA:pyruvate carboxylase	11	+	+	+	+	
Lsr2 family protein	11	+	+	+	+	
11 kD antitoxin	11		+	+		
Methylmalonyl-CoA carboxytransferase	11					+
Glycine cleavage system H protein	12	+	+	+	+	
Fe-S accesspry protein	12				+	
Uncharacterized protein	13	+				
Thioredoxin	13		+	+	+	+
DoxX family protein	13		+			+
Translation initiation inhibitor/YjgF	14-15		+			
Response regulator transcription factor	15			+		
PTS EIIA type 2 (sugar transporter)	15				+	
UPF0336	15		+		+	

**Table S2.** Amino acid sequences of the assigned proteins of *Cutibacterium acnes* subsp. *acnes* JCM 6425^{T}. Protein name	Gene name	Amino acid sequence	Molecular weight	Assigned m/z
50S ribosomal protein L7/L12	*rplL*	MAKLSNEELLDAFKEMTLIELSEFVKQFEETFDVSAAAPVA VAAAGAPAAGGEAAPAEEEKDEFDVILESAGDKKIQVIKEV RGLTSLGLKDAKDLVESAPKPVLEKAKKEDAEKAKEALEA AGATVTLK	13701.59	-Met $[M+H]^{+}$: 13571.41<br>-Met $[M+2H]^{2+}$: 6786.21
Lsr2-like protein	---	MAQRVRVDLVDDVDGSPAEESVNFALDGVNYVIDLSAEN ASKLRDALSLWVDHARRTGGRRTRGRRPTGGPTANEVR QWAKSQGYEVSERGRVSNEIREAYQRTH	11781.95	-Met $[M+H]^{+}$: 11651.76<br>-Met $[M+2H]^{2+}$: 5826.39
10 kDa chaperonin GroS	*groS*	VATTIKPLEDRVLVQPLEAEQTTASGLVIPDTAKEKPQEGK VISAGPGRVDDKGTRVPMDVKEGDVVIFSKYGGTEVKYD GQEYLLLNARDILAVVEK	10596.09	-Val $[M+H]^{+}$: 10497.97<br>-Val $[M+2H]^{2+}$: 5249.49
DNA-binding protein HU	*hup*	MTKSELIKTVAKQASLTEAQTNEAVKALTDVITAALAKGEK VQLPGLFTAETVERPARNGRNPRTGESMTIPAHKAVKISA SSTLKKAVAD	9588.06	$[M+H]^{+}$: 9589.07<br>$[M+2H]^{2+}$: 4795.04
CsbD-like protein	---	VGLSDKINSKSDEAVGAAKEKIGGLTDDSDLKSAGADQKA SGKVAQKVEDVKDKANDLKHNVQAAADKLKG	7278.07	-Val $[M+H]^{+}$: 7179.95<br>-Val $[M+2H]^{2+}$: 3590.48
7 kD antitoxin	---	MGLFDKAKDAISDRQDDIKNQASQHSDQVEQGIDKAGNT VDDKTGGKFSDQIDKGQDALKDKLGDL	7164.76	-Met $[M+H]^{+}$: 7034.57<br>-Met $[M+2H]^{2+}$: 3517.79
DUF3117 domain-containing protein	---	MAAMKPRTGDGPMEVTKEGRGIIMRVPVEGGGRLVVELN ADEAQELLACFKDVVG	5842.82	-Met $[M+H]^{+}$: 5712.64

Table S3. Comparison chart of observed prominent MALDI peaks and calculated masses of significant proteins in *Cutibacterium* spectra. acn IA_1: *C. acnes* subsp. *acnes* type IA_1, acn IB: *C. acnes* subsp. *acnes* type IB, acn II: *C. acnes* subsp. *defendens* type II, acn III: *C. acnes* subsp. *elongatum* type III, mod: *C. modestum*, nam: *C. namnetense*, avi: *C. avidum*, gra: *C. granulosum*. Obs.: Observed m/z. Calc.: Calculated m/z. ND: Not detected. NP: Not present. Underlines or double underlines indicate the same calculated *m/z* values across species and subspecies.

Protein name		Species/subspecies							
		acn IA_1	acn IB	acn II	acn III	mod	nam	avi	gra
Putative sheep haemoglobin	Calc.	15033.17	15033.17	15033.17	15033.17	15033.17	15033.17	15033.17	15033.17
alpha chain -Met $[M+H]^+$	Obs.	*15041.93*	*15047.06*	*15045.41*	*15045.67*	ND	*15041.64*	*15052.29*	*15046.48*
50S ribosomal prot L7/L12 -Met $[M+H]^+$	Calc.	13571.41	NP	13571.41	13626.48	13586.42	13529.37	13572.39	13602.42
	Obs.	*13565.43*	ND	ND	ND	*13583.00*	ND	13575.42	*13601.29*
Lsr2-like prot -Met $[M+H]^+$	Calc.	11651.76	11651.76	11651.76	11651.76	11712.76	11724.81	11629.67	11582.72
	Obs.	*11646.05*	*11649.39*	*11651.38*	*11648.23*	*11708.91*	ND	*11623.58*	*11581.71*
10 kD Chaperonin GroS -Val $[M+H]^+$	Calc.	10497.97	10497.97	10497.97	10497.97	10510.02	10497.97	10525.98	10497.97
	Obs.	*10493.82*	*10496.39*	*10495.79*	*10496.60*	*10507.86*	ND	ND	*10495.91*
DNA-binding prot HU $[M+H]^+$	Calc.	9589.07	9589.07	9589.07	9589.07	9529.96	9529.02	9514.99	9601.09
	Obs.	*9586.01*	*9587.65*	*9587.41*	*9589.36*	*9528.55*	*9527.97*	*9518.68*	*9600.35*
Putative sheep haemoglobin	Calc.	7517.09	7517.09	7517.09	7517.09	7517.09	7517.09	7517.09	7517.09
alpha chain -Met $[M+2H]^{2+}$	Obs.	*7523.20*	ND	*7523.96*	ND	*7524.36*	*7524.85*	*7527.10*	*7524.27*
CsbD-like prot -Val/-Met $[M+H]^+$	Calc.	7179.95	7179.95	7251.03	7237.98	7424.24	7283.97	7369.07	NP
	Obs.	*7179.01*	*7179.61*	*7250.54*	*7238.00*	*7424.35*	*7285.33*	*7372.75*	ND
7 kD Antitoxin -Met $[M+H]^+$	Calc.	7034.57	7004.55	6985.50	6971.52	6986.53	6940.46	7043.62	7017.54

	Obs.	*7033.48*	*7002.77*	*6985.48*	*6969.34*	*6986.52*	*6939.36*	*7047.01*	*7017.81*
50S ribosomal prot L7/L12 -Met $[M+2H]^{2+}$	Calc.	6786.21	NP	6786.21	6813.75	6793.72	6765.19	6786.70	6801.72
	Obs.	*6785.03*	ND	*6786.62*	ND	*6793.46*	ND	ND	*6801.65*
Lsr2-like prot -Met $[M+2H]^{2+}$	Calc.	5826.39	5826.39	5826.39	5826.39	5856.89	5862.91	5815.34	5791.87
	Obs.	*5825.25*	*5826.89*	*5825.46*	*5824.34*	*5856.20*	ND	*5813.98*	*5791.43*
DUF3117 domain-containing protein	Calc.	5712.64	5712.64	5712.64	5754.72	5692.65	5708.65	5678.58	5686.60
-Met $[M+H]^{+}$	Obs.	*5712.45*	*5711.39*	5710.68	*5753.77*	*5692.28*	ND	5681.95	*5686.97*
10 kD Chaperonin GroS -Val $[M+2H]^{2+}$	Calc.	5249.49	5249.49	5249.49	5249.49	5255.52	5249.49	5263.50	5249.49
	Obs.	*5248.94*	*5248.13*	*5247.38*	*5248.20*	*5255.26*	ND	ND	*5250.86*
DNA-binding prot HU $[M+2H]^{2+}$	Calc.	4795.04	4795.04	4795.04	4795.04	4765.48	4765.01	4758.00	4801.05
	Obs.	*4793.57*	*4792.28*	*4793.29*	*4794.08*	*4764.56*	*4762.25*	*4760.70*	*4801.21*
CsbD-like prot -Val/-Met $[M+2H]^{2+}$	Calc.	3590.48	3590.48	3626.02	3619.50	3655.58	3584.95	3627.50	NP
	Obs.	*3587.20*	*3587.20*	*3622.35*	*3616.04*	ND	ND	ND	ND
7 kD Antitoxin -Met $[M+2H]^{2+}$	Calc.	3517.79	3502.78	3493.26	3486.27	3493.77	3470.74	3522.32	3509.28
	Obs.	*3513.32*	*3497.88*	*3489.62*	*3496.78*	*3490.74*	ND	ND	*3508.86*

Table S4. Amino acid sequences of the assigned proteins of *Staphylococcus aureus* CCUG 1800^T. a: Assignment was made by referring to *Cutibacterium* assignment, b: to analysis by Becher *et al*. [11], c: to *S10-spc-alpha* operon gene list, d: to comprehensive genome analysis.

Protein name	Gene name	Amino acid sequence	Molecular weight	Assigned m/z
50S ribosomal protein L7/L12 [b]	*rplL*	MANHEQIIEAIKEMSVLELNDLVKAIEEEFGVTAAAPVAVA GAAGGADAAAEKTEFDVELTSAGSSKIKVVKAVKEATGLG LKDAKELVDGAPKVIKEALPKEEAEKLKEQLEEVGATVELK	12711.55	-Met $[M+H]^+$: 12580.36
30S ribosomal protein S15 [d]	*rpsO*	MAISQERKNEIIKEYRVHETDTGSPEVQIAVLTAEINAVNEH LRTHKKDHHSRRGLLKMVGRRRHLLNYLRSKDIQRYRELI KSLGIRR	10608.25	-Met $[M+H]^+$: 10478.07
30S ribosomal protein S16 [d]	*rpsP*	MAVKIRLTRLGSKRNPFYRIVVADARSPRDGRIIEQIGTYN PTSANAPEIKVDEALALKWLNDGAKPTDTVHNILSKEGIMK KFDEQKKAK	10234.86	-Met $[M+H]^+$: 10104.68
DNA-binding protein HU [a]	*hup*	MNKTDLINAVAEQADLTKKEAGSAVDAVFESIQNSLAKGE KVQLIGFGNFEVRERAARKGRNPQTGKEIDIPASKVPAFK AGKALKDAVK	9626.01	$[M+H]^+$: 9627.02<br>$[M+2H]^{2+}$: 4814.02
CsbD-like protein [a]	---	MADESKFEQAKGNVKETVGNVTDNKNLENEGKEDKASG KAKEFVENAKEKATDFIDKVKGNKGE	7018.69	-Met $[M+H]^+$: 6888.51<br>-Met $[M+2H]^{2+}$: 3444.76
50S ribosomal protein L32 [d]	*rpmF*	MAVPKRRTSKTRKNKRRTHFKISVPGMTECPNCGEYKLS HRVCKNCGSYNGEEVAAK	6484.54	-Met $[M+H]^+$: 6354.36
5.5 kD hypothetical protein [d]	---	MSFMDKAKDAVEKFKNSDNEQVKNVKDKINEYTGSNNEE KKENEDKEK	5655.18	-Met $[M+H]^+$: 5525.00
Glycopeptide resistance-associated protein GraF [d]	*graF*	MSNENQNKKAAEKAKEVEEKLKDKKEEKTEDINQTKQDIQ DTLN	5162.67	-Met $[M+H]^+$: 5032.49

50S ribosomal protein L36 [c]	*rpmJ*	MKVRPSVKPICEKCKVIKRKGKVMVICENPKHKQRQG	4305.36	$[M+H]^+$: 4306.37

Table S5. Comparison chart of observed prominent MALDI peaks and calculated masses of significant proteins in *Staphylococcus* spectra. aur: *S. aureus*, capi capi: *S. capitis* subsp. *capitis*, capi urea: *S. capitis* subsp. *urealiticus*, capr: *S. caprae*, epi: *S. epidermidis*, hom: *S. hominis*, lug: *S. lugdunensis*. Obs.: Observed m/z. Calc.: Calculated m/z. ND: Not detected. NP: Not present. Underlines or double underlines indicate the same calculated *m/z* values across species and subspecies.

Protein name		Species/subspecies						
		aur	capi capi	capi urea	capr	epi	hom	lug
Putative sheep haemoglobin	Calc.	15033.17	15033.17	15033.17	15033.17	15033.17	15033.17	15033.17
alpha chain -Met $[M+H]^+$	Obs.	*15042.92*	*15046.25*	*15043.91*	*15044.56*	*15042.32*	*15041.76*	*15043.53*
50S ribosomal prot L7/L12 -Met $[M+H]^+$	Calc.	12580.36	12533.27	12533.27	12533.27	12519.25	12532.23	12475.18
	Obs.	*12577.10*	*12530.38*	ND	*12531.30*	ND	*12526.23*	ND
30S ribosomal prot S15 -Met $[M+H]^+$	Calc.	10478.07	10477.08	10477.08	10478.07	10507.11	10521.14	10409.99
	Obs.	*10479.53*	*10474.45*	*10473.15*	*10480.77*	*10501.76*	*10515.16*	*10409.12*
30S ribosomal prot S16 -Met $[M+H]^+$	Calc.	10104.68	10192.69	10192.69	10229.76	10229.76	10172.74	10217.85
	Obs.	*10101.38*	*10193.03*	*10190.21*	*10226.78*	*10227.07*	*10106.73*	*10213.89*
DNA-binding prot HU $[M+H]^+$	Calc.	9627.02	9641.09	9641.09	9627.02	9627.02	9656.04	9643.07
	Obs.	*9624.58*	*9639.03*	*9639.62*	9625.35	*9625.37*	*9653.78*	*9640.08*
Putative sheep haemoglobin	Calc.	7517.09	7517.09	7517.09	7517.09	7517.09	7517.09	7517.09
alpha chain -Met $[M+2H]^{2+}$	Obs.	*7523.76*	*7524.89*	*7524.35*	*7524.22*	*7522.63*	*7522.85*	*7522.40*
CsbD-like prot -Met $[M+H]^+$	Calc.	6888.51	6711.31	6711.31	7045.60	6681.33	6476.11	7355.01
	Obs.	*6888.04*	*6711.29*	*6712.02*	*7045.60*	*6679.67*	*6473.92*	*7353.52*
50S ribosomal prot L32 -Met $[M+H]^+$	Calc.	6354.36	6371.39	6371.39	6385.41	6398.41	6398.41	6371.39

	Obs.	*6353.70*	*6370.44*	*6371.43*	*6385.44*	*6396.83*	*6396.33*	*6370.26*
5.5 kD Hypothetical prot -Met $[M+H]^+$	Calc.	5525.00	5098.48	5098.48	5213.52	5338.87	5237.68	5165.57
	Obs.	*5524.85*	*5098.60*	*5098.86*	*5213.87*	*5337.31*	*5236.04*	*5164.67*
Glycopeptide resistance-	Calc.	5032.49	5041.45	5041.45	5014.38	5112.57	4564.92	4953.38
. associated prot GraF –Met $[M+H]^+$	Obs.	*5032.46*	*5041.61*	*5041.85*	*5015.48*	*5111.36*	*4563.08*	*4952.56*
DNA-binding prot HU $[M+2H]^{2+}$	Calc.	4814.02	4821.05	4821.05	4814.02	4814.02	4829.03	4822.04
	Obs.	*4813.80*	*4822.65*	*4821.11*	*4812.46*	*4813.13*	*4827.57*	*4821.12*
50S ribosomal prot L36 $[M+H]^+$	Calc.	4306.37	4292.34	4292.34	4292.34	4292.34	4292.34	4292.34
	Obs.	*4305.95*	*4292.22*	*4292.43*	*4292.43*	*4290.37*	*4290.72*	*4291.35*
CsbD-like prot -Met $[M+2H]^{2+}$	Calc.	3444.76	3356.16	3356.16	3523.31	3341.17	3238.56	3678.01
	Obs.	*3443.31*	*3356.11*	*3355.13*	*3522.30*	*3338.78*	*3236.21*	*3676.55*

Figure S1. Identification of ORFs encoding GraF in *Staphylococcus* strains. A. Flanking region of the *GraF* gene. The *GraF* gene is located between two ORFs encoding 'Hypothetical protein' and 'ECF transporter S component' in the genome of DSM 20231^T. The GenBank IDs for the genomic sequences and the locations of the predicted *GraF* gene in the strains, DSM 6717^T, NCTC 12196^T, and NCTC 11320^T, were shown below. B. An alignment of predicted amino acid sequences of GraF. Sequence similarity scores (%) to the GraF protein in DSM 20231^T were shown right.

A

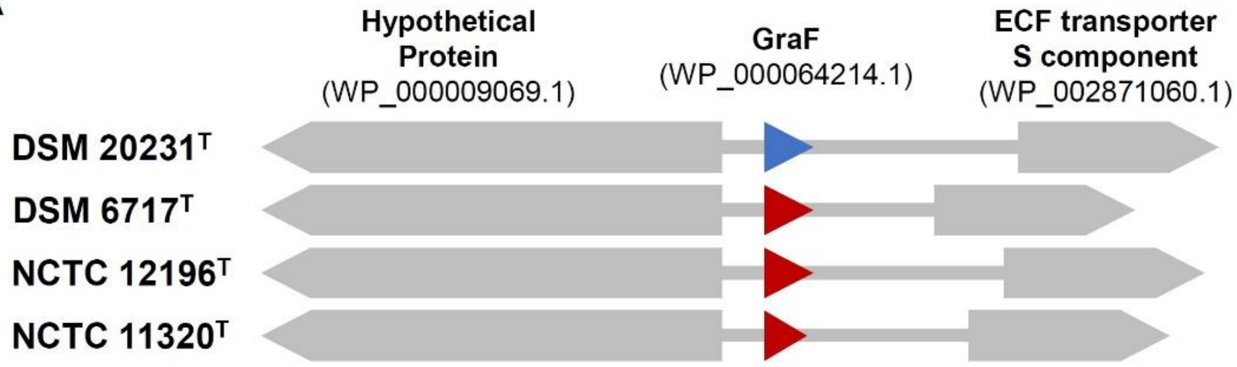

Strain	GenBank ID	Location
DSM 6717:	NZ_PPQI01000027	complement (25943.. 26077)
NCTC 12196	NZ_PPRT01000040	complement (26118.. 26252)
NCTC 11320	NZ_PPQE01000025	complement (397350..397469)

B

```
DSM 20231       MSNENQNKKAAEKAKEVEEKLKDKKEEKTEDINQTKQDIQDTLN
AYP 1020        MGKEEFEKKSAEKAKEAEEKLKEQNEEKTDDIEQTKKDVQDTLD 65 %
DSM 6717        MGKEEFEKKSAEKAKEAEEKLKEQNEEKTDDIEQTKKDVQDTLD 65 %
NCTC 12196      MGKEEFEKKSAEKAKEAEEKLKEQNEEKSNDTEQTKKDVQDTLD 61 %
ATCC 14990      MTNKEFEKKSAEKAKEVEEKLKEQNEEKTDDIEQTKKDIQDTLD 70 %
NCTC 11320      MTHED-----AKKAKEYEEKLKEQKEEKTDDIEQTKQDVQNTLD 64 %
NCTC 12217      MSNEELNKKSAEKAKEAEEKLKKEKESTEDSIEQTKKDVQDTLD 65 %
                *         * **** *****   *       *** * * **
```

Figure S2. Comparison of MALDI-TOF spectra of *Cutibacterium* and *Staphylococcus* species growing on agar plates with and without sheep blood. All materials were prepared on day 5 under otherwise the same culture conditions. Green boxes indicate presence of ca. *m/z* 15045 peaks with sheep blood in the agar plates.

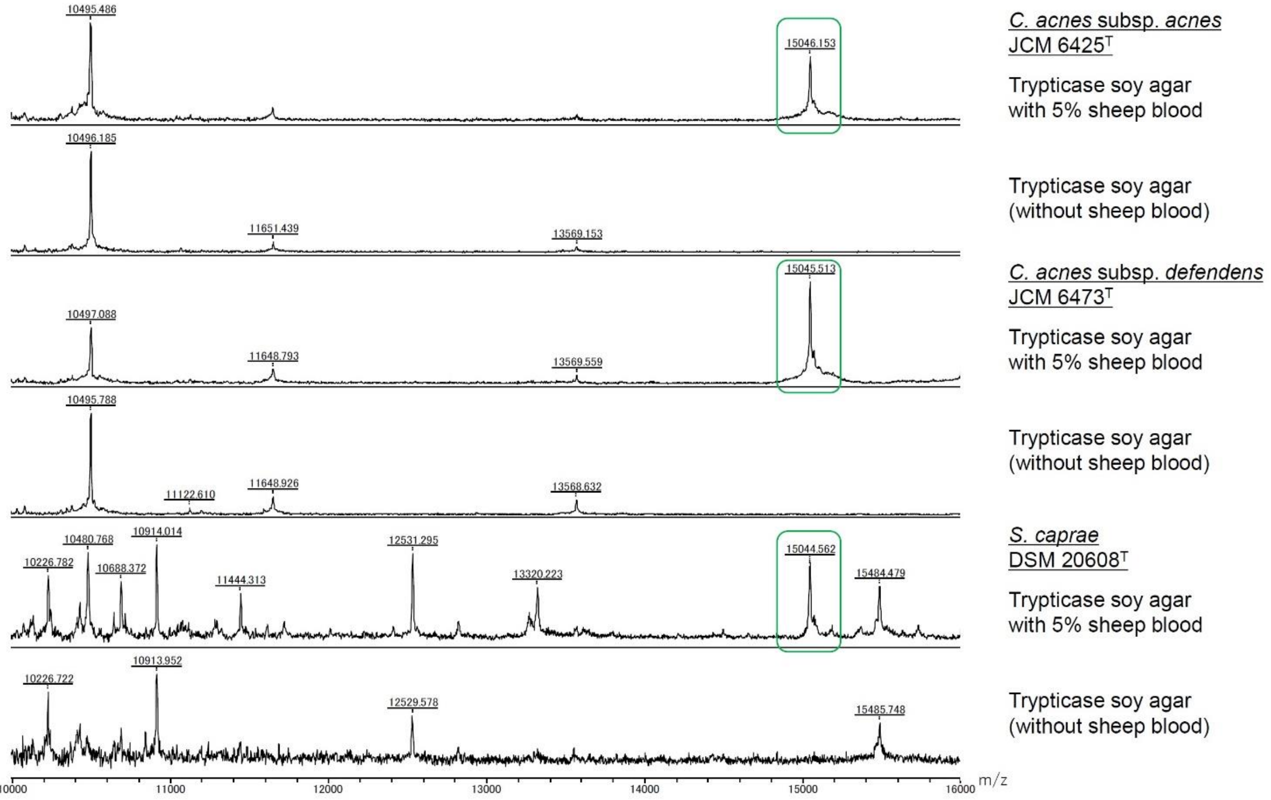

Supplement: Supplementary file 1 [file microorganisms-09-01243-s001.zip › microorganisms-1220010-supplementary.pdf]
